# Methane-Rich Saline Suppresses ER-Mitochondria Contact and Activation of the NLRP3 Inflammasome by Regulating the PERK Signaling Pathway to Ameliorate Intestinal Ischemia‒Reperfusion Injury

**DOI:** 10.1007/s10753-023-01916-0

**Published:** 2023-10-29

**Authors:** Zeyu Li, Ben Wang, Lifei Tian, Bobo Zheng, Xu Zhao, Ruiting Liu

**Affiliations:** Department of General Surgery, Shaanxi Provincial People’s Hospital, Xi’an, Shaanxi 710068 People’s Republic of China

**Keywords:** Methane saline, Intestinal ischemia‒reperfusion injury, Mitochondria-associated membranes, Inflammasome

## Abstract

Intestinal ischemia‒reperfusion (I/R) injury is a common pathological process in patients undergoing gastrointestinal surgery, leading to local intestinal damage and increased microvascular permeability, eventually causing extraintestinal multiple organ dysfunction or sepsis. The NLRP3-mediated inflammatory response is associated with I/R injury. Methane saline (MS) has anti-pyroptosis properties. This study aims to explore the protective effect of MS on intestinal I/R injury and its potential mechanisms. After MS pretreatment, the *in vivo* model was established by temporarily clipping the mouse superior mesentery artery with a noninvasive vascular clamp, and the *in vitro* model was established by OGD/R on Caco-2 cells. The results of HE and TUNEL staining showed intestinal barrier damage after I/R injury, which was consistent with the IHC staining results of tight junction proteins. Moreover, the expression of the NLRP3 signaling pathway was increased after I/R injury, and inhibition of NLRP3 activation reduced Caco-2 cell injury, indicating that NLRP3-mediated pyroptosis was one of the main forms of cell death after I/R injury. Subsequently, we found that MS treatment ameliorated intestinal barrier function after I/R injury by suppressing NLRP3-mediated pyroptosis. MS treatment also reduced mitochondria-associated membrane (MAM) formation, which was considered to be a platform for activation of the NLRP3 inflammasome. Importantly, MS reduced ER stress, which was related to the PERK signaling pathway. Knocking down PERK, a key protein involved in ER stress and MAM formation, reversed the protective effect of MS, indicating that MS suppressed NLRP3 by reducing ER stress and MAM formation. In conclusion, we believe that MS suppresses MAMs and activation of the NLRP3 inflammasome by regulating the PERK signaling pathway to ameliorate intestinal I/R injury.

## INTRODUCTION

Intestinal ischemia‒reperfusion injury (I/R) is a common pathophysiological injury after gastrointestinal surgery, trauma, volvulus, intestinal vascular thrombosis, or incarcerated hernia [1]. By disrupting the intestinal mucosal barrier and increasing microvascular permeability, I/R injury promotes bacterial and inflammatory cell infiltration, exacerbating local and systemic inflammatory responses, ultimately leading to multiple organ dysfunction or sepsis [2, 3]. Due to the complex mechanism of intestinal I/R injury, only limited therapies are currently available.

Pyroptosis is a newly discovered form of programmed cell death associated with inflammasome activation. As a well-known inflammasome, NOD-, LRR-, and pyrin domain-containing 3 (NLRP3)-mediated pyroptosis can not only occur in macrophages but also be identified in other types of cells during several stress conditions [4]. When the NLRP3 protein is activated by external stimuli, it combines with apoptosis-associated speck-like protein containing a CARD (ASC) and precursors of caspase-1 (pro-caspase-1) into the NLRP3 protein complex, the so-called NLRP3 inflammasome. Subsequently, the activation of the NLRP3 inflammasome transforms pro-caspase-1 into cleaved caspase-1, and caspase-1 then converts pro-IL-1β and pro-IL18 into mature IL-1β and IL-18. The cleaved caspase-1 then cleaves gasdermin (GSDM) family proteins, exposing their N-terminal structure, which can form pores on the surface of cell membranes to promote the release of mature IL-1β and IL-18 and lead to pyroptosis [5, 6]. Therefore, it is necessary to determine the occurrence of pyroptosis in intestinal I/R injury to improve the intestinal barrier in the pathological process.

As the site of protein folding in cells, the endoplasmic reticulum (ER) is vulnerable to stress conditions such as ATP depletion or hypoxia, resulting in the accumulation of misfolded or unfolded proteins. Under this condition, the unfolded protein response (UPR) is activated. The UPR is a signaling pathway mainly regulated by PKR-like ER kinase (PERK), inositol-requiring enzyme 1 (IRE1), and activated transcription factor 6 (ATF6), and its role is mainly to repair ER homeostasis within an appropriate range and promote cell survival [7]. However, excessive stress exceeds UPR regulatory capacity, and the inflammatory and apoptotic signaling pathways in cells are activated [8]. ER stress is a major reason for the pathological mechanisms underlying intestinal I/R injury [9]. ER stress can also induce the activation of the NLRP3 inflammasome [10].

Mitochondrial-associated ER membranes (MAMs) are a special membrane area between mitochondria and the ER that is involved in calcium ion exchange, lipid transfer, and metabolism [11]. A recent study showed that PERK is an important component protein on MAMs, and its activation can change the structure of MAMs, leading to cell apoptosis [12]. Suppression of the PERK/ATF4/CHOP pathway can not only inhibit ER stress but also remodel the structure of MAMs. Meanwhile, MAMs can serve as a platform for inflammatory signals, participating in the recruitment and release of NLRP3 and the occurrence of pyroptosis [13]. In response to external stimuli, NLRP3 is recruited together with the ASC protein to MAMs, driving the assembly and maturation of inflammasomes and thereby mediating the secretion of inflammatory factors [14]. Therefore, we hypothesize that ER stress induces NLRP3-mediated pyroptosis by increasing MAM formation.

Our previous studies have shown that MS can suppress the NLRP3 signaling pathway to alleviate sepsis-induced intestinal injury [15]. We also confirmed that MS can inhibit ER stress by regulating the GRP78/ATF4/CHOP signaling pathway [16]. However, studies on whether methane can protect against intestinal I/R injury remain scarce. In this study, we explored the therapeutic effect and underlying mechanisms of MS on intestinal I/R injury to provide a novel treatment for intestinal I/R injury.

## MATERIALS AND METHODS

### Experimental Animals

Healthy male C57BL/6 mice (7 weeks old, weighing 22–25 g) were purchased from the Animal Center of Xi’an Jiaotong University. The mice were housed for 7 days under standard conditions to adapt to the environment before initiation of the experiment. The procedures of animal experiments were performed according to the Animal Care guidelines of the China Council and were approved and supervised by the Institutional Animal Care and Ethics Committee (IACEC) of Xi’an Jiaotong University, China.

### The Preparation of Methane Saline

The methane saline and medium were prepared as described in a previous study [15]. In brief, methane was dissolved in 0.9% saline or DMEM (glucose-free) medium under pressure (0.4 MPa) for 4 h to a supersaturated concentration (1.2–1.5 mmol/L in saline and 1.4–1.8 mmol/L in DMEM) [17].

### Experimental Design

Mice were anesthetized by an intraperitoneal (i.p.) injection of 2% isoflurane. The intestinal I/R injury model was performed as follows. The superior mesenteric artery (SMA) was exposed by aparotomy and temporarily occluded with an atraumatic vascular clamp for 30, 60, and 90 min, and then the clamp was released to recover the blood supply for 120 min (*n* = 15). Among them, 10 mice continued to be raised for 72 h to evaluate the survival rate, while the other 5 mice were used to collect the intestinal tissue and blood samples for further tests. To confirm the protective effect of MS, another set of mice was randomly allocated into the following groups: Sham group, control group, and MS group (*n* = 5). MS (10 mL/kg) was intraperitoneally administered as a pretreatment during the reperfusion period.

The Caco-2 cell line was purchased from Shanghai Institute of Biochemistry and Cell Biology. Oxygen–glucose deprivation/reperfusion (OGD/R) was used to establish an I/R model *in vitro*. In brief, the Caco-2 cell line was cultured with glucose-free medium and kept in an incubator chamber (Phcbi, Japan, MCO-5 M) under certain conditions (95% N2 and 5% CO2) for 6 h to establish the hypoxia model and cultured in glucose-containing medium under certain conditions (5% O2, 5% CO2 for 2 h) for 2 h to establish the reoxygenation model [18]. The cells in the MS group were cultured in methane-rich medium with or without glucose. Caco-2 cells were incubated with CY-09 (10 μM) to suppress the expression of NLRP3.

### Histologic Analysis and TUNEL Assay

For the histological analysis, intestinal tissues were collected 120 min after reperfusion. Samples were fixed in 10% formalin and paraffin embedded and then sectioned into 5-μm-thick sections and stained with hematoxylin and eosin (H&E). Intestine injury scores were calculated as described in a previous study [19].

Intestinal tissues were assessed with TUNEL staining according to the manufacturer’s instructions. The sections were observed with a fluorescence microscope, and representative fields were chosen for application.

### Inflammatory Cytokine Assay

The levels of interleukins (IL-1β, IL-6) and tumor necrosis factor (TNF-α) in intestinal tissues and the levels of I-FABP in serum were measured by ELISA kits (Nanjing Jiancheng, China).

### Transepithelial Electrical Resistance

The intestinal mucosae were collected as soon as the mice were executed, and then transepithelial electrical resistance (TER) was used to evaluate intestinal barrier integrity and detected with an Ussing chamber (EM-CSYS, PI, USA) as described [20].

### Western Blot Assay

Western blotting was used to detect protein expression in intestinal tissues as previously described [21]. Briefly, total protein and nucleoprotein were extracted with RIPA lysis buffer according to the manufacturer’s instructions. After the protein concentration was determined, protein samples were separated using 10–25% sodium dodecyl sulfate‒polyacrylamide gel electrophoresis (SDS‒PAGE) and transferred onto polyvinylidene difluoride (PVDF) membranes. Blocking of the resulting blots was performed using 8% skim milk and further incubated with anti-occludin antibody (1:1000; Proteintech, China), anti-ZO-1 antibody (1:1000; Proteintech, China), anti-NLRP3 antibody (1:1000; Proteintech, China), anti-Caspase1 antibody (1:1000; Abcam, USA), anti-IL-1β antibody (1:1000; Abcam, USA), anti-N-terminus of GSDMD (1:1000; Abcam, USA), anti-PERK antibody (1:1000; Proteintech, China), anti-GRP78 antibody (1:1000; Proteintech, China), anti-ATF4 antibody (1:1000; Proteintech, China), anti-CHOP antibody (1:1000; Proteintech, China), and anti-β-actin antibody (1:10,000; Santa Cruz Biotechnology, USA) overnight at 4 °C. Subsequently, the blots were incubated with anti-rabbit and anti-mouse horseradish peroxidase-conjugated secondary antibodies (1:10,000; Abcam, USA) for 1 h at 37 °C after washing three times with PBS. The proteins were detected with the chemiluminescence (ECL) system. The expression of proteins was normalized to β-actin as a reference.

### Immunohistochemistry Analysis

Immunohistochemistry staining was performed as previously described [22]. Anti-ZO-1 (1:400; Proteintech, China), anti-occludin (1:400; Proteintech, China), anti-NLRP3 (1:200; Proteintech, China), anti-caspase1 (1:100; Abcam, USA), anti-IL-1β (1:100; Abcam, USA), and anti-CHOP antibodies (1:400; Proteintech, China) were used to detect protein expression in the intestinal tissue. The slides were stained with diaminobenzidine tetrahydrochloride (DAB), counterstained with hematoxylin and mounted for microscopic examination.

### Immunofluorescence Staining and PI Staining

Immunofluorescence staining was performed as previously described [22]. Briefly, the cells and tissue sections were washed with PBS-0.25% Tween-20 (PBS-T) three times, blocked with 5% goat serum, and incubated with the primary antibodies rabbit anti-NLRP3 (1:200, CST, USA), caspase-1 (1:200, Abcam, USA), IL-1β (1:200, CST, USA), and PERK (1:200, Proteintech, China) overnight at 4 °C. Then, the cells and tissue sections were incubated with the fluorescent secondary antibody (goat anti-rat antibody or goat anti-rabbit antibody, Proteintech, China, diluted 1:100) for 1 h. Finally, the cells and tissue sections were counterstained with 4′-6-diamidino-2-phenylindole (DAPI) and observed with a fluorescence microscope. (Beyotime, China).

Pyroptosis was detected by propidium iodide (PI) staining following the manufacturer’s recommendations [23]. The quantification of fluorescence intensity was performed using ImageJ software.

### Cell Viability Assay and LDH Detection

Cell viability was evaluated with the Cell Counting Kit-8 (CCK-8, Abcam, USA) following the manufacturer’s instructions. The optical density (OD) values were measured at 450 nm.

Lactate dehydrogenase (LDH) activity was measured with a commercial kit (Nanjing Jiancheng, China) according to the manufacturer’s instructions.

### Knockdown of PERK in Caco-2 Cells

To knock down PERK, Caco-2 cells were transfected with 100 nM PERK target siRNA or control nonspecific siRNA (GeneChem, China) with lipofectamine 2000 reagent (GeneChem, China) following the manufacturer’s protocol. After 24 h, the cells were subjected to OGD/R as previously mentioned. The efficiency of depletion was confirmed by immunofluorescence staining.

### Transmission Electron Microscopic Analysis

The intestinal tissues were fixed in 2.5% glutaraldehyde to further observe the ultrastructure of MAM integrity of intestinal epithelial cells. Transmission electron microscopy (TEM) was used to observe all of the microscopic images. The aspect ratio of mitochondria and endoplasmic reticulum contact points was assessed using ImageJ software and calculated as previously described [24]. In brief, 50 pictures of each experimental group were obtained at × 20,000 magnification. The images were analyzed using ImageJ (National Institutes of Health). The mitochondrial and ER membranes were delineated using the freehand tool. The selected areas were converted to masks and the perimeters of ER were calculated. For the MAM quantification (distance between ER/SR and mitochondria within 30 nm), we normalized the total ER connected to mitochondria to total ER perimeter.

### Statistical Analysis

The measurement data were described as the mean ± standard deviation (SD). All statistical analyses were performed with SPSS18.0 software (SPSS Inc., USA). Student’s t test and one-way analysis of variance (ANOVA) were used for comparisons among the three groups. The figure was made by GraphPad (version 7) Prism software (GraphPad Software, CA). All tests were two sided, and significance was accepted at *p* < 0.05.

### I/R Injury-Induced Disruption of Intestinal Barrier Function

**Fig. 1:**
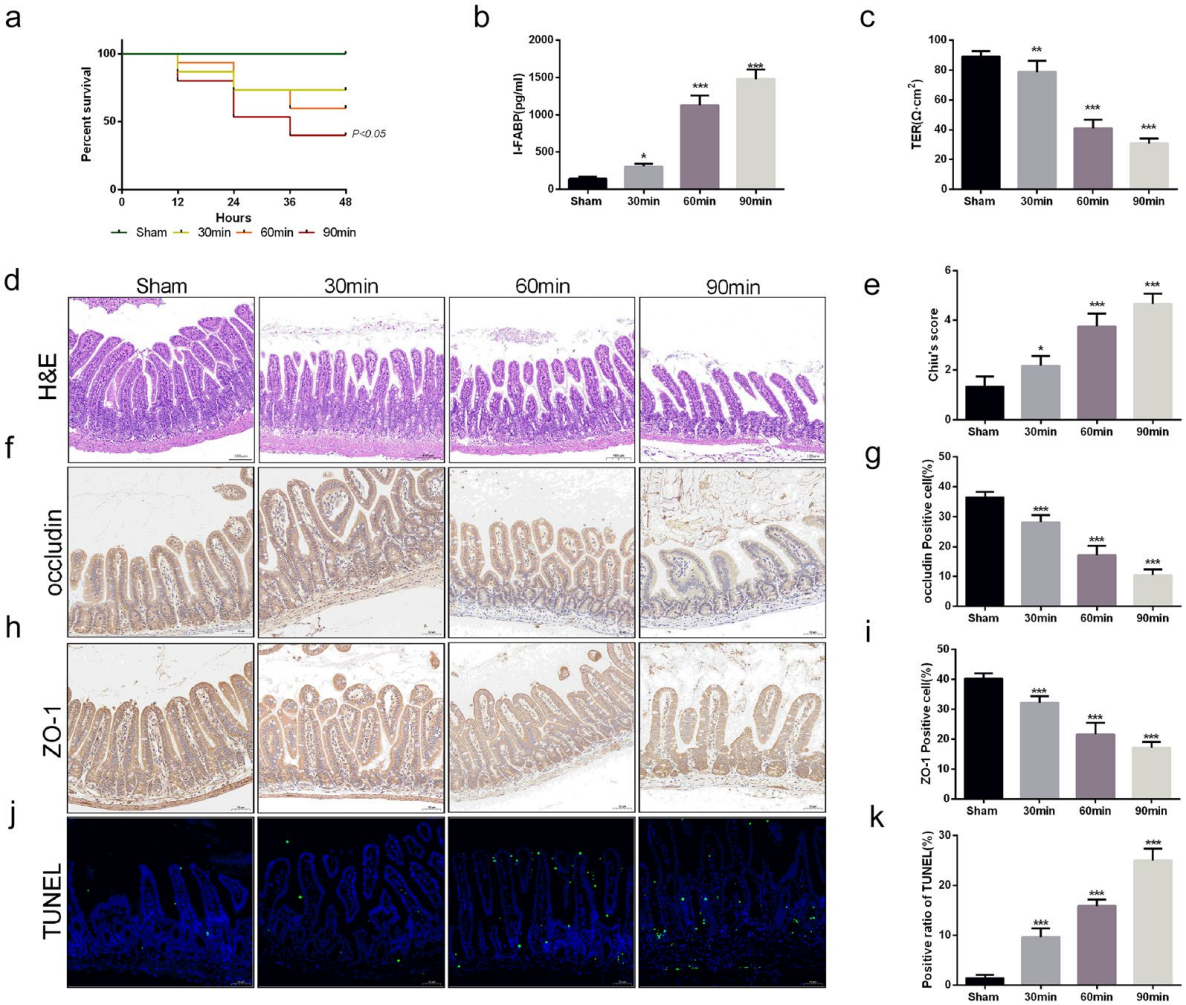
I/R injury-induced intestinal barrier disruption and epithelial cell injury. Mice were subjected to I/R or sham surgery and divided into four groups: sham, 30 min, 60 min, and 90 min (*n* = 5). Intestines were collected after ischemia. **a** Survival rates were calculated in different groups (*n* = 10). **b**, **c** The integrity of the intestinal barrier was evaluated with serum I-FABP levels and TER. **d**, **e** Histopathological damage was estimated with H&E staining (scale bars: 100 μm) and Chiu’s score. **f**–**i** The expression levels of ZO-1 and occludin were analyzed by IHC staining (scale bars: 50 μm). **j**, **k** Representative images of TUNEL staining of intestinal sections (scale bars: 50 μm). The values are shown as the mean ± SD. *p < 0.05, **p < 0.01, ***p < 0.001 compared with the sham group.

To detect the impact of intestinal I/R injury on intestinal barrier function in mice, we observed the survival rate after intestinal I/R in mice and found that the longer the ischemia time was, the lower the survival rate (Fig. 1a). The intestinal fatty binding protein (I-FABP) is specifically and abundantly present in epithelial cells of the mucosal layer of the small intestinal tissue. I-FABP is also considered to be rapidly released into the circulation just after small intestinal mucosal tissue is injured. Then, we detected the level of I-FABP in the serum (Fig. 1b). The results showed that the level of I-FABP gradually increased with prolonged ischemic time, indicating an obvious disorder of the intestinal barrier structure. The results of TER also showed that I/R injury induced a decrease in TER compared with that in the sham group (Fig. 1c). Moreover, we conducted a histopathological examination to detect the impacts on the intestinal barrier in mice after I/R injury (Fig. 1d, e). The results of H&E staining and histological scoring showed that the damage to the intestinal barrier was more severe with prolonged ischemic time, which was consistent with the results of I-FABP and TER. Then, the expression of ZO-1 and occludin, two tight junction proteins, was further analyzed to explore the adverse effects of I/R injury on intestinal integrity (Fig. 1f–i). The expression of these two tight junction proteins was also decreased with prolonged ischemic time. Consistent with the results of the tight junction protein, TUNEL staining also showed a higher positive cell rate after I/R injury (Fig. 1j, k). According to the results above, we demonstrated that the intestinal barrier is disrupted in a time-dependent manner after I/R injury.

### I/R Injury Induced NLRP3-Mediated Inflammatory Responses and MAM Increases in the Intestine

**Fig. 2:**
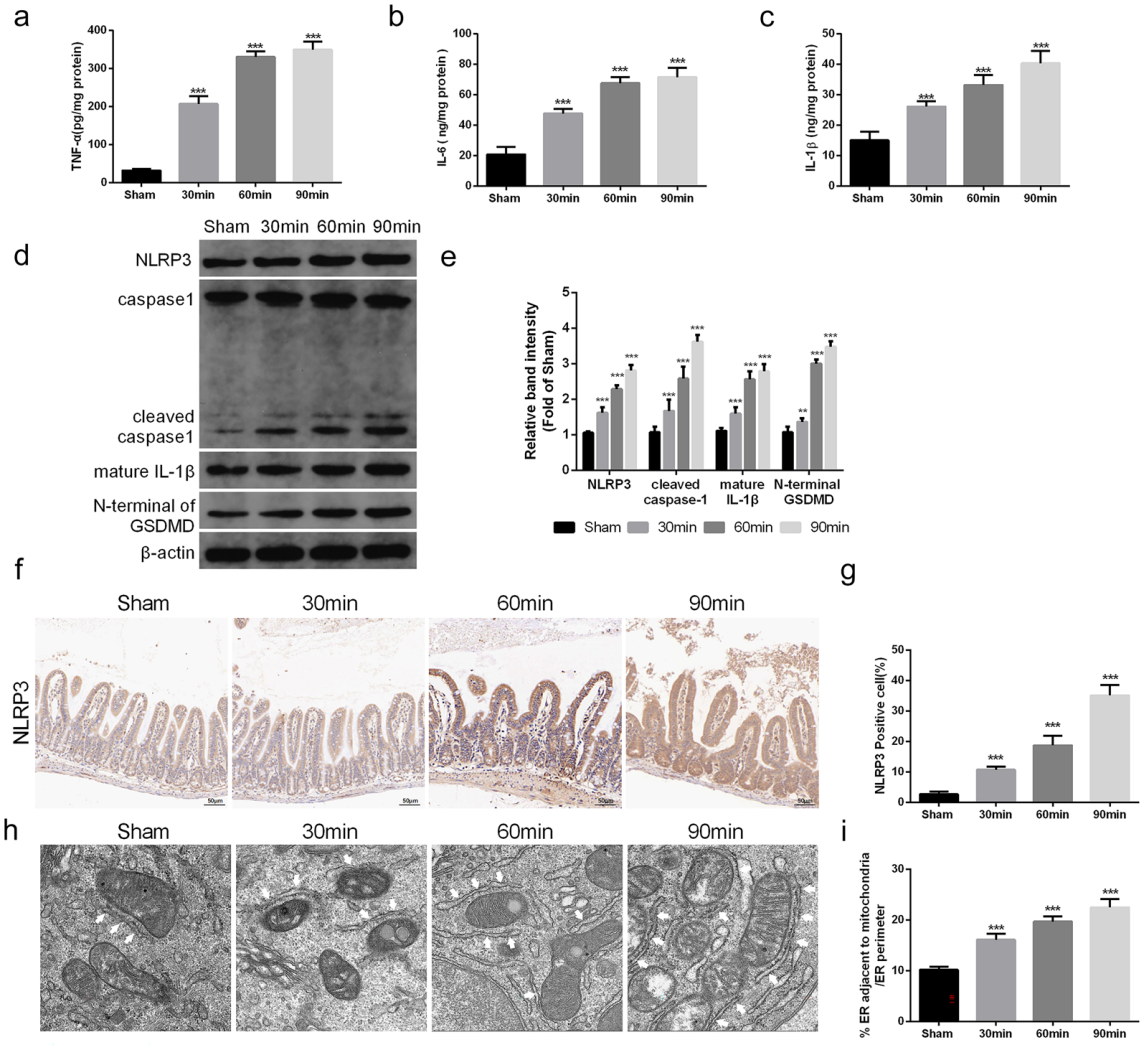
I/R injury-induced intestinal inflammation and activation of NLRP3-related pyroptosis. Mice were subjected to I/R or sham surgery and divided into four groups: sham, 30 min, 60 min, and 90 min (*n* = 5). The levels of TNF-α (**a**), IL-6 (**b**), and IL-1β (**c**) were evaluated by ELISA. **c**, **d** The NLRP3 signaling pathway, including NLRP3, caspase-1, IL-1β, and GSDMD, was measured by Western blotting. **f**, **g** The IHC staining of NLRP3 was used to examine the occurrence of pyroptosis (scale bars: 50 μm). **h**, **i** Representative TEM images of MAMs. The white arrow illustrates quantitation of ER length adjacent to mitochondria normalized by total ER length (scale bars: 500 nm). The values are shown as the mean ± SD. *p < 0.05, **p < 0.01, ***p < 0.001 compared with the sham group.

The inflammatory response is involved in the destruction of the intestinal barrier after I/R injury. Thus, we first measured the levels of inflammatory cytokines in intestinal tissue (Fig. 2a–c). As the ischemia time increased, the levels of TNF-α, IL-1β, and IL-6 in the intestinal tissue also gradually increased after I/R injury. Then, we determined whether the excessive inflammatory response after I/R injury was caused by pyroptosis. We detected the expression of the NLRP3 signaling pathway via Western blot assay (Fig. 2d, e). The results showed that the expression of NLRP3 was significantly increased after I/R injury, and the activation of caspase-1 and the downstream product IL-1β also revealed an evident increase *in vivo*. We also found that the expression of the N-terminus of GSDMD, the effector molecule of pyroptosis, was increased *in vivo* in an ischemic time-dependent manner, indicating that NLRP3-mediated pyroptosis was one of the major forms of cell death after I/R injury.

**Fig. 3:**
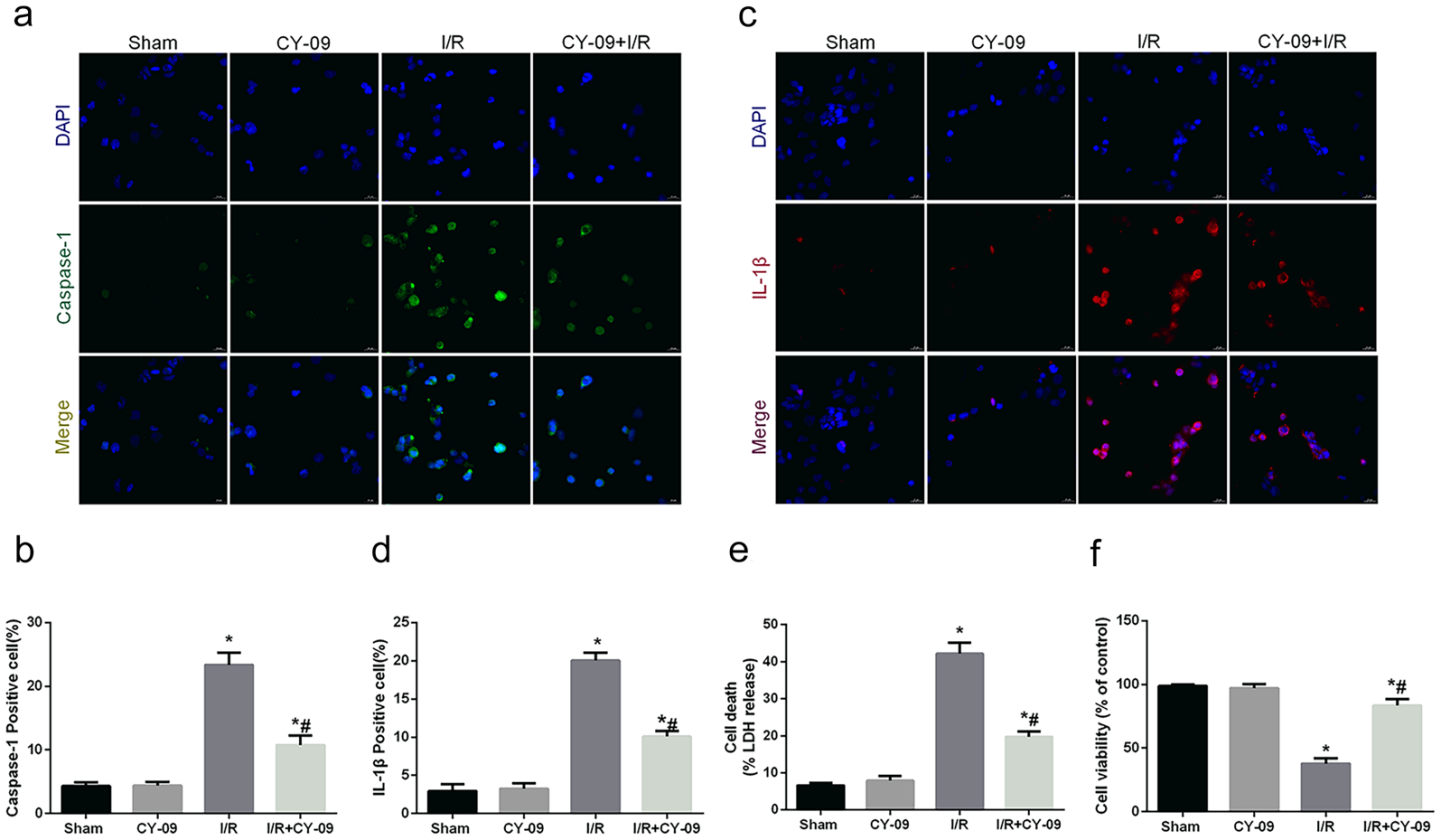
I/R injury-induced activation of NLRP3-related pyroptosis *in vitro*. The OGD/R model was established in Caco-2 cells, and CY-09 (10 μM), a specific inhibitor of NLRP3 inflammasomes, was used as a positive control. **a**–**d** The expression of caspase-1 and IL-1β was detected *in vitro* by immunofluorescence (scale bars: 20 μm). **e** The release levels of LDH were detected. **f** Cell viability was measured with a CCK-8 assay. The values are shown as the mean ± SD. ^*^p < 0.05, compared with the sham group, and ^#^*p* < 0.05 compared with the I/R group.

Then we used IHC staining to measure the expression of NLRP3 (Fig. 2f, g). The results showed that the longer the ischemic time was, the higher the NLRP3 positive rate in intestinal tissue, which was consistent with the Western blot results. Moreover, we assessed MAM formation, a platform for NLRP3 inflammasome activation, by analyzing the changes in the physical interface between the ER and mitochondria with transmission electron microscopy (Fig. 2h–i). As illustrated above, the physical interface between the ER and mitochondria was markedly increased after I/R injury. We also used CY-09 (10 μM) to inhibit the activation of NLRP3 in Caco-2 cells to explore the role of pyroptosis after I/R injury *in vitro*. The IF staining results revealed that the expression of caspase-1 and IL-1β was significantly increased after I/R injury, and this trend was reversed by inhibition of NLRP3 inflammasome activity (Fig. 3a–d). We also found that the release of LDH and cell viability were significantly improved after NLRP3 silencing (Fig. 3e, f), which was consistent with the results of IF staining. Taken together, these data revealed that NLRP3-mediated inflammatory responses and pyroptosis played an important role in intestinal I/R injury.

### Effect of MS on Intestinal Barrier Function During IR Injury

**Fig. 4:**
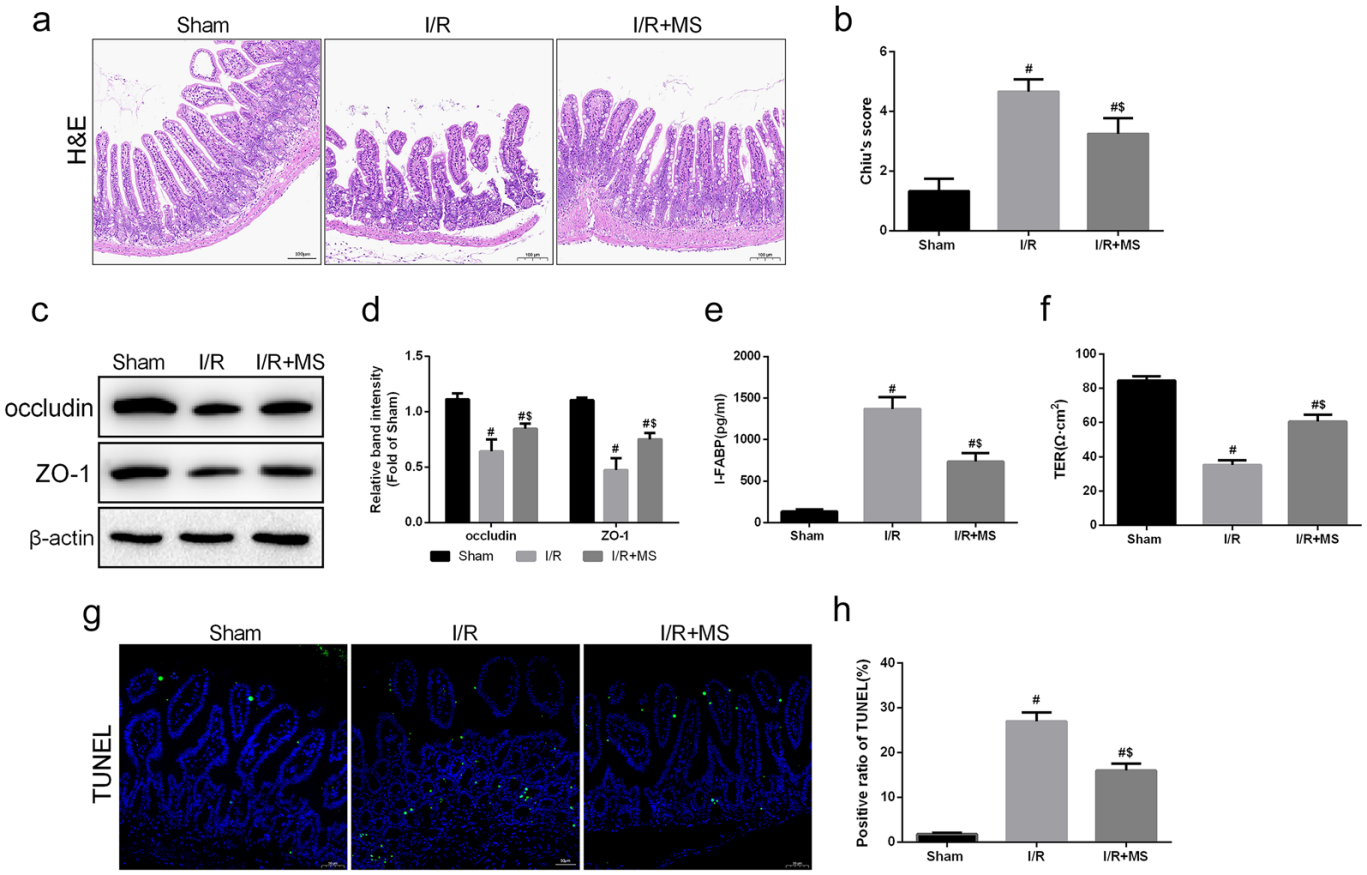
Methane-rich saline alleviated intestinal barrier disruption and epithelial cell injury. The animal models were launched with a 60-min ischemia period as mentioned. MS (10 mL/kg) was used to treat the mice as previously mentioned (*n* = 5). **a**, **b** Histopathological damage was estimated with H&E staining (scale bars: 100 μm) and Chiu’s score. **c**, **d** The expression of ZO-1 and occludin was analyzed by Western blotting. **e**, **f** The integrity of the intestinal barrier was evaluated with serum I-FABP levels and TER. **g**, **h** Representative images of TUNEL staining of intestinal sections (scale bars: 50 μm). The values are shown as the mean ± SD. ^#^*p* < 0.05, compared with the sham group, and ^$^*p* < 0.05 compared with the I/R group.

To determine the protective effect of MS, HE staining was used to evaluate the histopathological examination after I/R injury (Fig. 4a, b). The results revealed that MS treatment significantly reduced intestinal tissue damage after I/R injury. Then, we determined whether MS protected intestinal barrier function by measuring the expression of ZO-1 and occludin (Fig. 4c, d). The results showed that the expression of these two tight junction proteins was suppressed after I/R injury and that MS treatment effectively reversed this trend. MS treatment also decreased the levels of I-FABP and increased TER (Fig. 4e, f). In addition, the treatment of MS also showed significant TUNEL staining results. The results showed that TUNEL-positive cells significantly increased after I/R injury, and this increasing trend was inhibited by MS treatment (Fig. 4g, h). These data suggested that MS treatment alleviated intestinal I/R injury.

### MS Treatment Alleviated Intestinal I/R Injury by Inhibiting NLRP3-Mediated Pyroptosis and Increasing MAM Levels

**Fig. 5:**
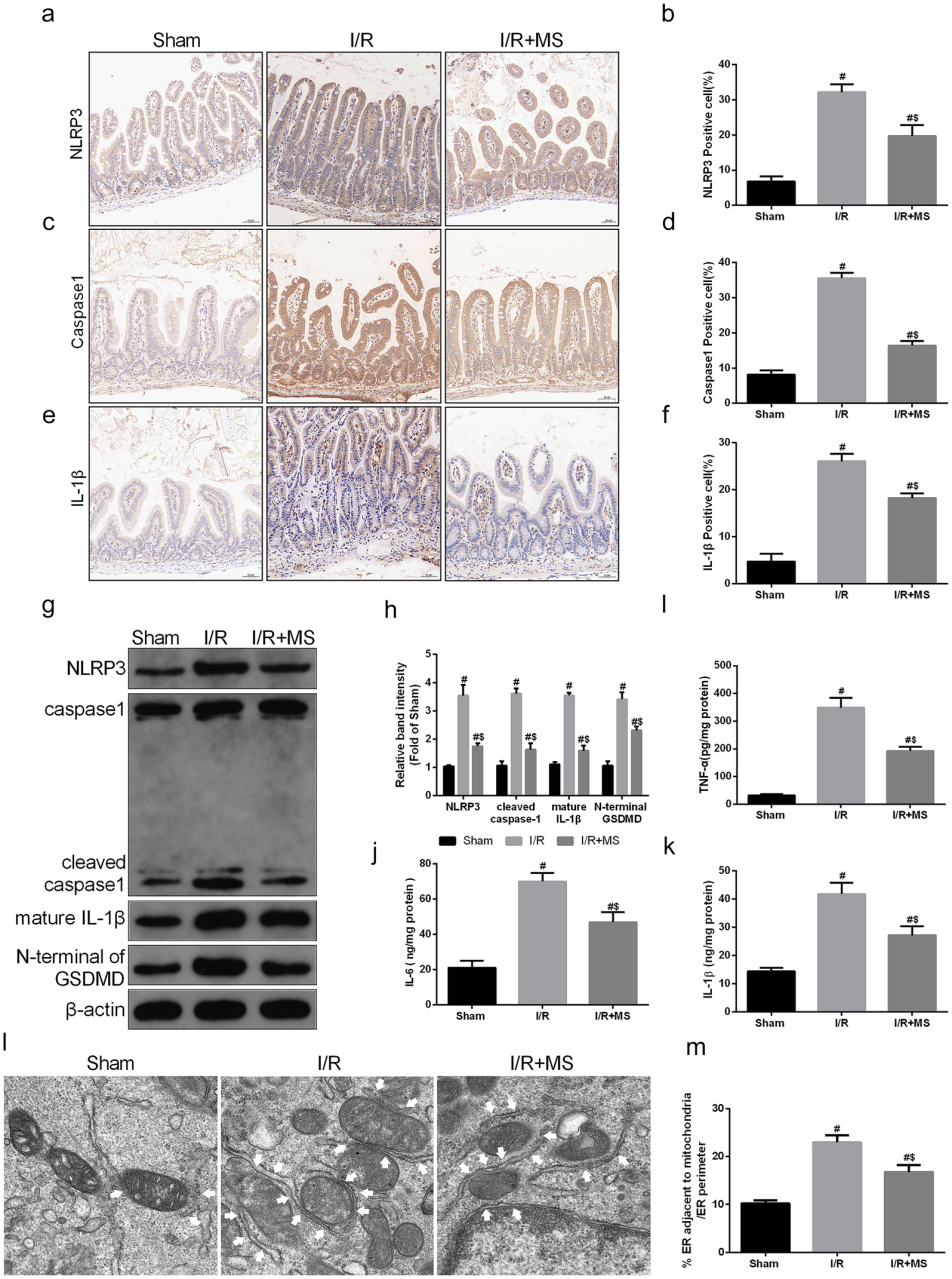
Methane-rich saline reduced NLRP3-related pyroptosis and MAMs. The animal models were launched with a 60-min ischemia period as mentioned. MS (10 mL/kg) was used to treat the mice as previously mentioned (*n* = 5). **a**–**f** Immunohistochemical staining of NLRP3, caspase-1, and IL-1β. **g**, **h** The NLRP3 signaling pathway, including NLRP3, caspase-1, IL-1β, and GSDMD, was measured by Western blotting. The levels of TNF-α (**i**), IL-6 (**j**), and IL-1β (**k**) were evaluated by ELISA. **l**, **m** Representative TEM images of MAMs. The white arrow illustrates quantitation of ER length adjacent to mitochondria normalized by total ER length (scale bars: 500 nm). The values are shown as the mean ± SD. ^#^*p* < 0.05, compared with the sham group, and ^$^*p* < 0.05 compared with the I/R group.

**Fig. 6:**
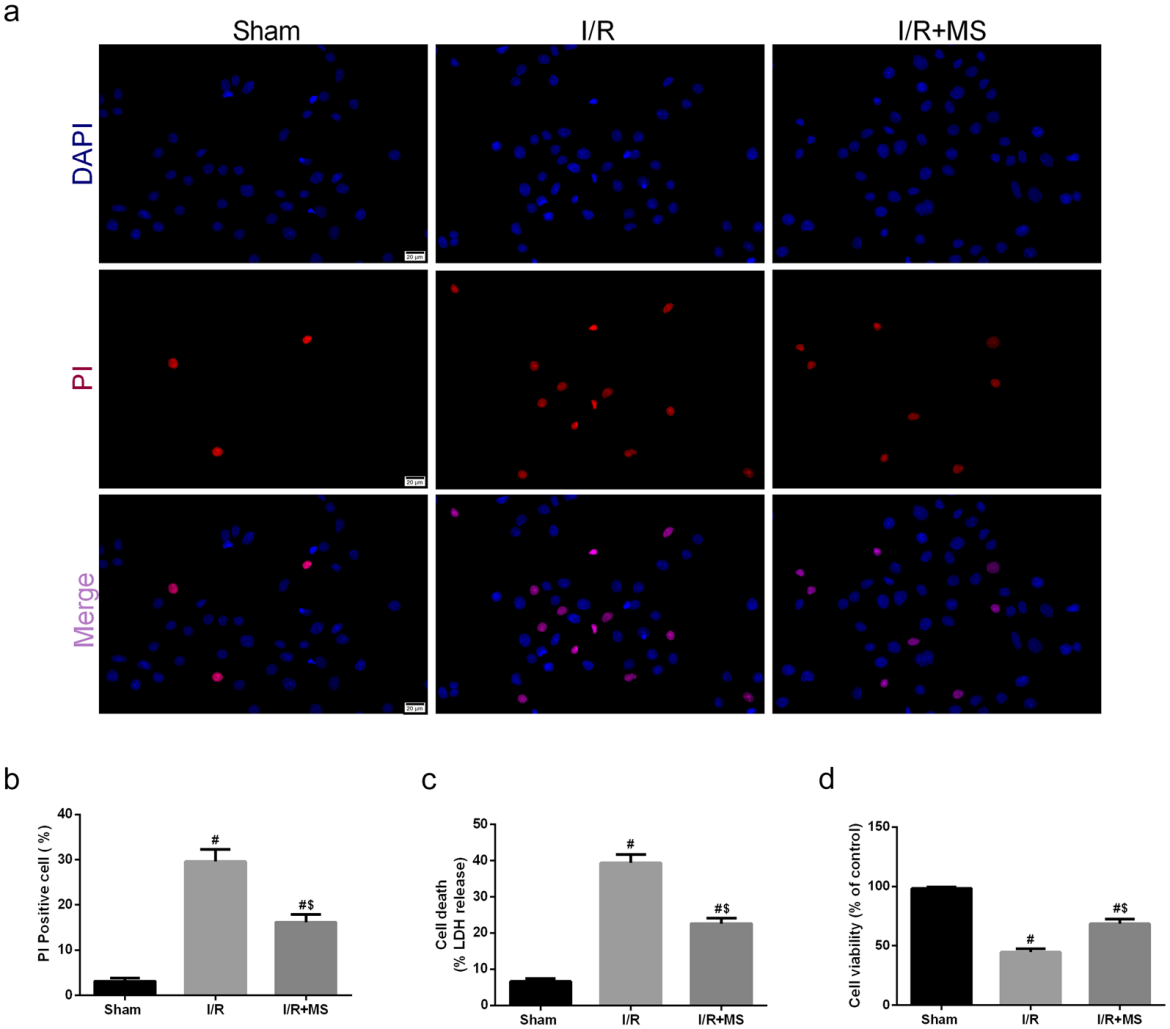
Methane-rich saline reduced NLRP3-related pyroptosis *in vitro*. The OGD/R model was established in Caco-2 cells, and MS was administered as previously mentioned. **a**, **b** PI staining was used to examine the occurrence of pyroptosis (scale bars: 20 μm). **c** The release levels of LDH were detected. **d** Cell viability was measured with a CCK-8 assay. The values are shown as the mean ± SD. ^#^*p* < 0.05, compared with the sham group, and ^$^*p* < 0.05 compared with the I/R group.

To detect whether MS protected against intestinal I/R injury by inhibiting the activation of the NLRP3 inflammasome. We used IHC staining to measure the expression of NLRP3 and caspase-1 and its downstream product IL-1β (Fig. 5a–f). The results showed that the number of NLRP3-positive cells dramatically increased after I/R injury, and this increasing trend was inhibited by MS treatment. The IHC results of caspase-1 and IL-1β obtained the same results. To further confirm this result, we measured the NLRP3 signaling pathway via Western blot assay (Fig. 5g, h). Consistent with the IHC results, the expression of NLRP3, caspase-1, IL-1β, and N-GSDMD was markedly increased after I/R injury. However, quantification of these protein expression levels exhibited a profound decrease after MS treatment. Next, we examined the levels of inflammatory cytokines in the different groups (Fig. 5i, k). MS treatment also decreased the levels of IL-6, IL-1β, and TNF-α after I/R injury. We also examined MAM formation via TEM (Fig. 5l–m). The results showed that MS treatment can reduce MAM formation. In addition, we explored the occurrence of pyroptosis in Caco-2 cells after I/R injury *in vitro*. The PI staining results showed that the fluorescence intensity in the I/R group was significantly increased, and this trend was reversed by MS treatment (Fig. 6a, b). The levels of LDH and cell viability were consistent with the PI staining results (Fig. 6c, d). Considering that MAMs are platforms for the activation of NLRP3 inflammasomes, we believe that MS treatment can inhibit pyroptosis by inhibiting the formation of MAMs based on the results above.

### MS Treatment Inhibited PERK-Mediated ER Stress to Reduce MAM Formation

**Fig. 7:**
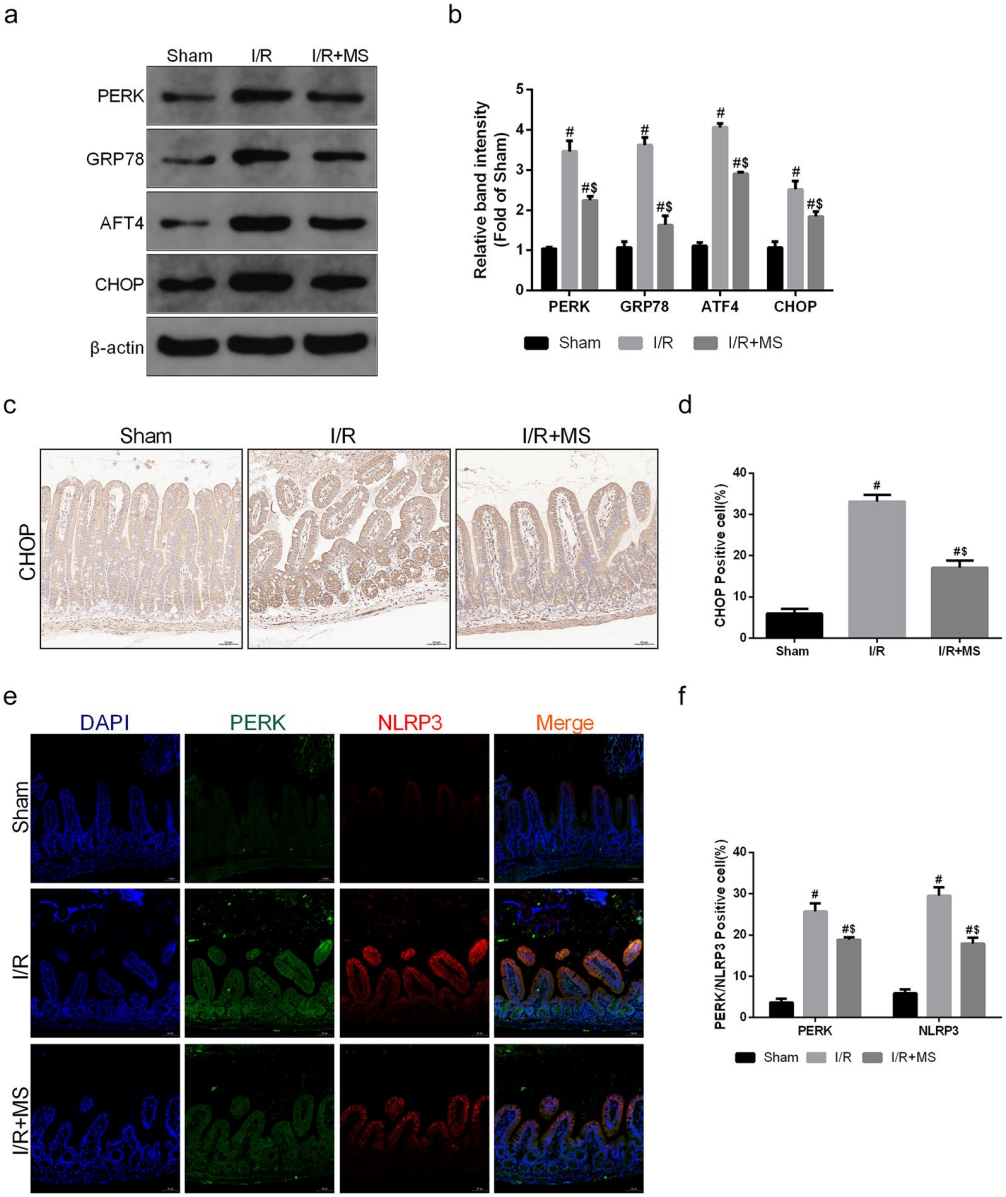
MS reduced pyroptosis and MAMs by inhibiting ER stress. The animal models were launched with a 60-min ischemia period as mentioned. MS (10 mL/kg) was used to treat the mice as previously mentioned (*n* = 5). **a**, **b** The expression of PERK, GRP78, ATF4, and CHOP was measured by Western blotting. **c**, **d** Immunohistochemical staining of CHOP (scale bars: 50 μm). **e**, **f** The colocalization of PERK and NLRP3 was shown by immunofluorescence (scale bars: 50 μm). The values are shown as the mean ± SD. ^#^*p* < 0.05, compared with the sham group, and ^$^*p* < 0.05 compared with the I/R group.

**Fig. 8:**
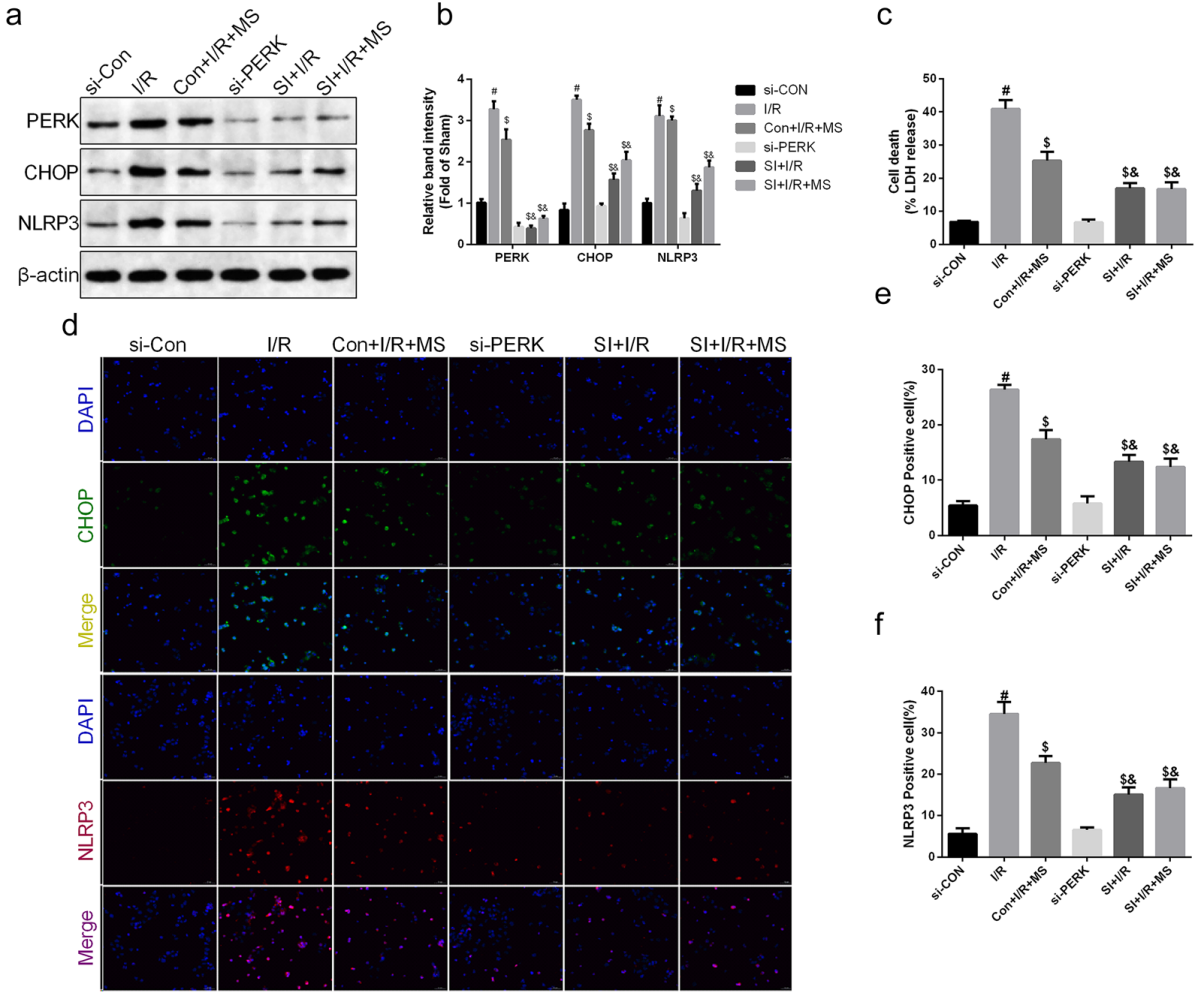
Knockdown of PERK abolished MS-inhibited pyroptosis *in vitro*. Caco-2 cells were transfected with PERK-targeting siRNA or control siRNA. Then, cells were induced as OGD/R models and received MS treatment as previously mentioned. **a**, **b** The expression of PERK, CHOP, and NLRP3 was measured by Western blotting. **c** The release levels of LDH were detected. **d**–**f** Immunohistochemical staining of CHOP and NLRP3 (scale bars: 50 μm). The values are shown as the mean ± SD. ^#^*p* < 0.05, compared with the si-Con group, ^$^*p* < 0.05 compared with the Con + I/R group, ^&^*p* < 0.05 compared with the Con + I/R + MS group.

As a key ER stress sensor of the unfolded protein response, PERK is uniquely enriched at MAMs [12]. Overactivation of PERK not only leads to the occurrence of ER stress but also promotes the formation of MAMs. To explore the mechanism of MS on ER stress, we measured the expression of the PERK signaling pathway via Western blot (Fig. 7a, b). The results showed that the expression of PERK/GRP78/ATF4/CHOP was increased after I/R injury. MS treatment suppressed the activation of the PAERK signaling pathway. We next detected the expression of CHOP in the intestine via IHC staining after I/R injury (Fig. 7c, d). MS treatment inhibited the expression of CHOP, which was consistent with the Western blot results. Finally, we determined the interaction of PERK-NLRP3 *in vivo* (Fig. 7e, f). The immunofluorescence colocalization results in the intestine showed that the interaction between PERK and NLRP3 strongly increased after I/R injury and that MS treatment decreased this interaction, indicating that MS inhibited NLRP3-mediated pyroptosis by suppressing the PERK signaling pathway. Therefore, we further silenced PERK in Caco-2 cells to explore the protective effect of MS on intestinal I/R injury in a PERK-dependent manner. Western blot showed that knockdown of PERK not only inhibited the expression of CHOP and NLRP3 but also reversed the protective effect of MS after I/R injury (Fig. 8a, b). MS treatment exhibited a similar effect as previous results in the groups transfected with control siRNA. However, MS treatment exhibited no significant difference in the PERK-silenced groups regarding I/R-induced inflammasome activity and pyroptosis. The levels of LDH in the SI + IR group and IS + IR + MS group were not different, which also indicated that knockdown of PERK reversed the protective effect of MS *in vitro* (Fig. 8c). The immunofluorescence experiment also showed the same results (Fig. 8d–f). These results suggested that MS treatment could suppress pyroptosis by downregulating the expression of the PERK signaling pathway and MAM formation, which reduced intestinal I/R injury.

## DISCUSSION

Considering the high incidence rate, intestinal ischemia‒reperfusion injury has long been a tough postoperative challenge for clinical surgeons [1]. Emergency gastrointestinal surgery is one of the common causes of intestinal IR injury, causing undetected and rapidly progressive injury, which limits the prevention of intestinal IR injury. Intestinal IR injury could further damage the intestinal barrier, thus leading to higher permeability and changes in the intestinal microbial environment, eventually causing a severe inflammatory response and organ dysfunction [25]. For these reasons, the development of diagnostic and therapeutic strategies for intestinal IR injury is urgently needed. Our previous studies have reported that methane possesses antioxidative and anti-inflammatory properties [15, 19, 22]. However, studies on intestinal IR injury remain scarce. In this study, we explored the protective effect of MS on intestinal I/R injury and its potential mechanism. First, we found that I/R injury leads to intestinal barrier disruption, which is related to the activation of NLRP3. Next, we examined the protective effect of MS on intestinal I/R injury, which is related to the suppression of NLRP3-mediated pyroptosis *in vivo* and *in vitro*. MAMs have been proven to be a platform for NLRP3-mediated inflammatory responses [13]. We also found that MS treatment reduced MAM formation during I/R injury. PERK is not only a protein-regulating ER stress but also enriched at MAMs [12]. Hence, we further explored whether MS inhibited ER stress after IR injury and the interaction between PERK and NLRP3. To examine our hypothesis, we silenced PERK and found that the protective effect of MS was reversed. According to these results, we concluded that MS suppresses MAM formation and activation of the NLRP3 inflammasome via the PERK signaling pathway to ameliorate intestinal I/R injury.

Few studies have revealed the role of the NLRP3-mediated inflammatory response in intestinal I/R injury. We demonstrated that the expression of NLRP3 increased in a time-dependent manner after I/R injury, while silencing the expression of NLRP3 effectively alleviated intestinal I/R injury, indicating that NLRP3-mediated pyroptosis occurred and was the main form of cell death after intestinal I/R injury. Recent studies have shown that MAMs play an important role in the activation of NLRP3 [13, 26]. Under a state of stimulation, the physical interaction of MAMs increases, and activated NLRP3 enters the MAM fractions and interacts with ASC and pro-caspase-1 to further mature the NLRP3 inflammasome, which is released into the cytosol to induce pyroptosis [27]. We examined whether MAMs increased during intestinal I/R injury, potentially indicating that MAMs also play an important role in NLRP3-mediated pyroptosis of the intestinal epithelium.

As a type of gas with small molecules possessing the proper distribution characteristics to penetrate membranes and diffuse into organelles, methane has been proposed to be a new therapeutic gas for sepsis-induced intestinal injury involving the inhibition of NLRP3-mediated pyroptosis [15]. However, the effect of MS on intestinal I/R injury remains unknown. Based on our study, MS effectively alleviated the intestinal barrier against I/R injury by reducing tight junction protein injury and maintaining the integrity of the intestinal barrier structure. Notably, MS treatment also inhibited the activation of the NLRP3 inflammasome and MAM increase. Thus, we believe that MS could suppress the NLRP3 signaling pathway to improve intestinal I/R injury.

Defined as functional and physical contact sites of mitochondria and the ER, MAMs play a key role in calcium signaling, lipid metabolism, inflammation, and so on. Acting as a stress sensor to monitor the condition of the ER, PERK has been confirmed to be located on MAMs [28]. Generally, in response to ER stress, PERK is activated by autophosphorylation and homomultimerization. After dissociation from GRP78, activated p-PERK further induces the activation of ATF4 and CHOP. In our study, the expression of PERK/GRP78/ATF4/CHOP was significantly increased, indicating that ER stress occurred due to intestinal I/R injury, and MS treatment downregulated the PERK signaling pathway to suppress ER stress. In addition, ER stress also promotes MAM increase [29]. Notably, MS treatment also reduced MAM formation. Silencing PERK abolished the protective effect of MS, indicating that MS suppresses MAM formation and ER stress to inhibit NLRP3-mediated pyroptosis to ameliorate intestinal I/R injury.

## CONCLUSION

In conclusion, we demonstrated that MS alleviated intestinal I/R injury and the mechanism, including regulating the PERK signaling pathway to reduce MAM increase and pyroptosis. MS might be a potential treatment option for intestinal I/R injury. However, there are still some limitations that need to be addressed in this study. Many prospective clinical trials are needed to confirm the therapeutic effect of MS. We believe that MS has considerable therapeutic potential in treating intestinal ischemia‒reperfusion injury.

## Data Availability

The data used to support the findings of this study are included in the paper.
